# Mapping disparities in homicide trends across Brazil: 2000–2014

**DOI:** 10.1186/s40621-020-00273-y

**Published:** 2020-09-07

**Authors:** Elaine Okanyene Nsoesie, Antonio S. Lima Neto, Jonathan Jay, Hailun Wang, Kate Zinszer, Sudipta Saha, Adyasha Maharana, Fatima Marinho, Adauto Martins Soares Filho

**Affiliations:** 1 Department of Global Health, School of Public Health, Boston University, Boston, MA USA; 2 Fortaleza Municipal Health Secretariat (SMS-Fortaleza), Fortaleza, Ceará Brazil; 3 University of Fortaleza (UNIFOR), Fortaleza, Ceará Brazil; 4 Takemi Program, Harvard T.H. Chan School of Public Health, Boston, MA USA; 5 Firearm-safety Among Children and Teens Consortium, University of Michigan School of Medicine, Ann Arbor, MI USA; 6 Department of Health Policy & Management, Harvard T. H. Chan School of Public Health, Boston, MA USA; 7 Department of Social and Preventive Medicine, University of Montreal, Montreal, Quebec, Canada; 8 Department of Global Health and Population, Harvard T. H. Chan School of Public Health, Boston, MA USA; 9 Department of Computer Science, University of North Carolina, Chapel Hill, NC, USA; 10 Department of Health Surveillance, Ministry of Health, Brasilia, Brazil

**Keywords:** Brazil, Homicide, Violent crimes

## Abstract

**Background:**

Homicides are a major problem in Brazil. Drugs and arms trafficking, and land conflicts are three of the many factors driving homicide rates in Brazil. Understanding long-term spatiotemporal trends and social structural factors associated with homicides in Brazil would be useful for designing policies aimed at reducing homicide rates.

**Methods:**

We obtained data from 2000 to 2014 from the Brazil Ministry of Health (MOH) Mortality Information System and sociodemographic data from the Brazil Institute of Geography and Statistics (IBGE). First, we quantified the rate of change in homicides at the municipality and state levels. Second, we used principal component regression and k-medoids clustering to examine differences in temporal trends across municipalities. Lastly, we used Bayesian hierarchical space-time models to describe spatio-temporal patterns and to assess the contribution of structural factors.

**Results:**

There were significant variations in homicide rates across states and municipalities. We noted the largest decrease in homicide rates in the western and southeastern states of Sao Paulo, Rio de Janeiro and Espirito Santo, which coincided with an increase in homicide rates in the northeastern states of Ceará, Alagoas, Paraiba, Rio Grande Norte, Sergipe and Bahia during the fifteen-year period. The decrease in homicides in municipalities with populations of at least 250,000 coincided with an increase in municipalities with 25,000 people or less. Structural factors that predicted municipality-level homicide rates included crude domestic product, urbanization, border with neighboring countries and proportion of population aged fifteen to twenty-nine.

**Conclusions:**

Our findings support both a dissemination hypothesis and an interiorization hypothesis. These findings should be considered when designing interventions to curb homicide rates.

## Background

Brazil faces a severe epidemic of lethal violence. In 2017, Brazil’s 63,880 homicides were the country’s highest total ever and represented a 3% increase from 2016 (Brazilian Forum on Public Security 2018). Within Brazil, geographical homicide patterns changed in recent decades. Homicide rates declined by 15% in Brazil’s Southeast region and increased by 20% in the Northeast from the early 1990s to late 2000s (Reichenheim et al. 2011). Earlier in that period, high homicide rates were predominantly associated with major metropolitan centers, particularly Rio de Janeiro and São Paulo (Steeves et al. 2015). However, by the mid-2010s, these cities were not among the leaders in homicide rates. In 2017, homicide rates in smaller cities such as Rio Branco (84 per 100,000), Fortaleza (77 per 100,000) and Belem (68 per 100,000) substantially outpaced Rio de Janeiro (33 per 100,000) and Sao Paulo (11 per 100,000) (Brazilian Forum on Public Security 2018).

Considering their public health significance, these changes in the spatial distribution of Brazil’s lethal violence have received only modest study. Waiselfisz and colleagues described the changes in terms of two processes: dissemination, by which violence moved from one state to another, and interiorization, by which violence moved from more urban to less urban locations within states (Waiselfisz 2012). Their analysis, however, did not attempt to quantify or compare these phenomena.

Subsequent studies have supported these hypotheses using either spatial or longitudinal analysis. For dissemination, Ingram and da Costa found evidence across most Brazilian regions for spatial dissemination, i.e. positive association in homicide rates among neighboring municipalities, but only using cross-sectional data from a single year (Ingram 2017). By contrast, Peres and Nivette used longitudinal methods and found that steep increases in a small number of municipalities explained a large proportion of the nationwide homicide increase from 1991 to 2010 (Peres and Nivette 2017). These increases were associated with baseline levels of social disorganization, a theory focused on how ecological conditions (such as, social structural conditions) can compromise community-level social control. The authors proposed that higher social disorganization could have made Brazilian cities more vulnerable to violence either through social disorganization or association with inadequate or repressive policing. However, these analyses did not account for spatial relationships among municipalities.

For interiorization, Andrade and Diniz used spatial analysis, but no formal statistical testing, to propose that economic development in less urban areas predicted the upward trend in homicides (Andrade and Diniz 2013). Steves and colleagues using state panel data and longitudinal models, found evidence to support the claim that violence was “following the money” within states to less populated areas (Steeves et al. 2015).

In other words, prior research supports the dissemination and interiorization hypotheses, but none has used methods suitable for evaluating space-time patterns*.* Such patterns are central to each hypothesis. The existing literature suggests important changes in homicide trends both between and within states, some of which may be explained by social structural conditions such as economic development and social disorganization. It remains unknown, however, the extent to which spatial relationships influence these trends. Evidence of dissemination, either related or unrelated to social structural conditions, would tend to support regionally targeted interventions to address violence. In contrast, lack of a dissemination pattern would tend to support macroeconomic policy changes and other large-scale interventions. Additional potential drivers are drug trafficking gangs and land conflicts resulting from deforestation and changes in agricultural practices (Barcellos and Zaluar 2014a; Tavares et al. 2016).

The purpose of this study was to contribute to the understanding of how Brazil’s national homicide rates remain critically high despite substantial declines in many locations. First, we aimed to describe changes in homicide trends across small and large municipalities in Brazil. Despite much discussion, changes in homicide rates had not been adequately measured by municipalities, and states over time. Second, we aimed to quantify the magnitude of spatial and temporal trends in homicides across Brazilian municipalities and the extent to which social structural conditions can explain them. Unlike previous studies, we employed a hierarchical Bayesian spatiotemporal model that explicitly accounted for trends in both time and space, as well as the nested structure of data collected from municipalities within states. The advantages of the Bayesian approach include: the ability to specify a prior which can be based on expert opinion or previous studies on the topic; the representation of uncertainty in the posterior distribution which captures the boundaries of a parameter and is therefore more intuitively interpretable compared to the *p*-value; and the ease of capturing the hierarchical structure present in the data.

## Methods

### Data

Data on homicides was obtained from the Brazil Ministry of Health’s Mortality Information System (Sistema de Informação de Mortalidade (SIM)). The data consisted of all-cause homicides reported by the police and was available for each municipality for each year from 2000 to 2014. We also obtained population estimates from the 2000 and 2010 census, and projections for all the other years from the Brazilian Institute of Geography and Statistics (IBGE). Additionally, sociodemographic and geographic data were also obtained from IBGE. These included municipality-level covariates (i.e., gross domestic product, the proportion of the population aged 15 to 29), and state-level illiteracy rate. Dichotomous variables (i.e., yes/no response) at the municipality level included whether a municipality shares borders with another country (hereafter referred to as, borders country strip) or located near other municipalities which share a border with a neighboring country (hereafter referred to as, borders country area/zone), and whether a municipality is a metro region. These variables were obtained for each year of the study period. While the relationship of these factors (such as, those related to social conditions and inequality) with crime have been studied in Latin American countries, further studies are needed to understand how homicide rates have changed over time and space in relation to these factors (Briceño-León et al. 2008; Dare et al. 2019; Heinemann and Verner 2006; Moser and McIlwaine 2006; Willman and Makisaka 2019). Baseline characteristics of the 26 states and the Federal District are presented in Table 1.

**Table 1:** Baseline characteristics of the 26 Brazilian states and the Federal District from the 2010 census. The table includes total population, population by sex, income, age and education

State	Population			Income	Age			Education		
	Total	Male	Female	Per capita income	Aged 0–24 years	Aged 25–49 years old	Aged 50 and over	% aged 25 or older graduated from elementary school	% aged 25 or older graduated from secondary school	% aged 25 or older graduated from higher education
Acre	733,559	368,324	365,235	522.15	53.79	33.5	12.72	43.98	31.56	8.98
Alagoas	3,120,494	1,511,767	1,608,727	432.56	48.5	34.77	16.73	36.36	24.98	6.9
Amapá	669,526	335,135	334,391	598.98	54.32	34.66	11.02	57.89	43.89	10.84
Amazonas	3,483,985	1,753,179	1,730,806	539.8	53.4	34.19	12.41	52.07	37.77	8.23
Bahia	14,016,906	6,878,266	7,138,640	496.73	44.39	36.72	18.9	41.75	29.82	6.4
Ceará	8,452,381	4,120,088	4,332,293	460.63	45.64	35.47	18.89	42.88	29.23	7.16
Distrito Federal	2,570,160	1,228,880	1,341,280	1715.11	41.83	41.97	16.2	69.85	55.4	23.95
Espírito Santo	3,514,952	1,731,218	1,783,734	815.43	40.86	38.52	20.61	50.64	36.09	11.06
Goiás	6,003,788	2,981,627	3,022,161	810.97	42.13	39.4	18.48	50.06	34.69	10.27
Maranhão	6,574,789	3,261,515	3,313,274	360.34	51.3	32.85	15.84	38.53	26.36	5.43
Mato Grosso	3,035,122	1,549,536	1,485,586	762.52	44.48	38.99	16.53	48.29	33.03	10.47
Mato Grosso do Sul	2,449,024	1,219,928	1,229,096	799.34	43.19	37.67	19.14	49.36	34.88	11.99
Minas Gerais	19,597,330	9,641,877	9,955,453	749.69	40.04	37.68	22.28	46.4	32.25	10.57
Pará	7,581,051	3,821,837	3,759,214	446.76	51.32	34.67	14.01	43.53	28.51	6.21
Paraíba	3,766,528	1,824,379	1,942,149	474.94	43.87	35.68	20.46	37.67	26.98	8.02
Paraná	10,444,526	5,130,994	5,313,532	890.89	40.42	38.02	21.56	50.85	35.62	12.75
Pernambuco	8,796,448	4,230,681	4,565,767	525.64	44.2	36.5	19.3	43.05	30.59	8.01
Piauí	3,118,360	1,528,422	1,589,938	416.93	45.96	34.9	19.14	35.92	24.47	7.29
Rio de Janeiro	15,989,929	7,625,679	8,364,250	1039.3	37.27	38.29	24.45	62.04	44.45	14.31
Rio Grande do Norte	3,168,027	1,548,887	1,619,140	545.42	43.97	36.96	19.07	43.93	31.57	8.32
Rio Grande do Sul	10,693,929	5,205,057	5,488,872	959.24	37.18	37.22	25.6	52.14	35.43	11.28
Rondônia	1,562,409	795,157	767,252	670.82	46.83	37.88	15.29	42.68	29.02	8.04
Roraima	450,479	228,859	221,620	605.59	53.16	34.8	12.04	54.3	40.97	10.16
Santa Catarina	6,248,436	3,100,360	3,148,076	983.9	39.58	39.28	21.14	53.78	37.03	12.53
São Paulo	41,262,199	20,077,873	21,184,326	1084.46	38.3	39.42	22.29	59	42.33	15.1
Sergipe	2,068,017	1,005,041	1,062,976	523.53	46.37	36.67	16.95	42.5	30.29	8.53
Tocantins	1,383,445	702,424	681,021	586.62	48.37	35.51	16.12	46.78	34.45	10.25

### Statistical analysis

#### Exploratory spatial analysis

To describe changes in homicide rates across the country, we first calculated and mapped state- and municipality-level homicide rates at the beginning and the end of the study period. We also estimated the percent change over time for homicide rates per 100,000 persons within 10 years and assessed changes both at the municipality and state level.

#### Temporal analysis using principal components clustering

Our first major aim was to assess changes in homicide rates at small and largely populated municipalities over time. One of our hypotheses was that lethal violence rose in smaller cities as drug trafficking expanded in search of an alternative internal consumer market due to the economic growth of the “Lula’s years” (2003–2010). The Atlas da Violência made this paradoxical connection between economic growth and increased violence in the cities of the Northeast and North Regions after years of economic stagnation (PÚBLICA Anuário Brasileiro de Segurança 2017).

To test this hypothesis, we compared the trajectories in homicide rates observed in smaller municipalities to the trajectories observed in larger ones over time using principal components regression and clustering. First, we stratified the municipalities into four population sizes based on 2014 population estimates; up to 25,000, 25,001-100,000, 100,001-250,000, and above 250,000. Next, we applied principal components cluster analysis (PCCA) to each subgroup to further separate the municipalities into clusters based on the reported temporal trajectories of the homicide rates. The homicide rates for the years 2000 to 2014 for each municipality was represented as a time series; *y*_*i*_ = [*y*_*i*_(*t*_1_), *y*_*i*_(*t*_2_), …, *y*_*i*_(*t*_*n*_)] where the *y*_*i*_ and t represent homicide rate and time, respectively (Campbell et al. 2006; Jones and Rice 1992). The time series curves represented an n-dimensional vector; therefore, we could compute principal components from the variance covariance matrix of the following matrix: *Y* = [*y*_1_, *y*_2_, …, *y*_*n*_] where n is the number of municipalities in the subgroup. We clustered the regression coefficients resulting from the model fitted to the principal components since these were projections onto the principal component axis, which indicated the major structure of the homicide time series curves. Additionally, few coefficients were needed for clustering when using a non-scaled clustering algorithm such as, Partition Around Medoids (PAM) (Kaufman and Rousseeuw 1990), which we applied in this study. The variance of the regression coefficients declined with increase in the number of principal components. We used the first n (i.e., 4, 8, 11 and 14, respectively) principal components for each population group, which explained approximately 96% of the variance, in clustering. Increasing the number of principal components did not improve the clustering outcomes. We explored different numbers of clusters and decided on three to four clusters with the expectation that municipalities will be grouped as follows: increasing, decreasing, stagnant and/or no obvious trend.

#### Hierarchical Bayesian space-time model

Our second major aim was to investigate spatial and temporal variation in homicide rates, while incorporating sociodemographic and geographic features. Unlike previous studies, we employed a hierarchical Bayesian spatiotemporal model that explicitly accounted for trends in both time and space, as well as the nested structure of data collected from municipalities within states. The annual homicide rate in each municipality was modeled as a binomial random variable with each covariate independently and with various combinations of covariates due to significant correlations between covariates (e.g., the correlation between crude domestic product and illiteracy was − 0.706).

Homicide rates and change in homicide rates might be more similar in neighboring municipalities than in those more distant, which did not follow the independent and identically distributed (iid) assumptions in non-spatial models. We used the Moran’s I statistic to test for spatial autocorrelation. Furthermore, we used the Besag spatial model (Besag et al. 1991), that included spatially structured random effects that follow a distribution conditional on neighboring municipalities homicide rates. Structured random effects accounted for the variation in homicide rates that was due to the spatial ordering of the municipalities. Unstructured random effects accounted for unobserved heterogeneity that was not explained by the spatial effects or the sociodemographic and geographical factors. This enabled the estimation of homicide rates for each municipality based on the data for that municipality, and data for neighboring municipalities, and accounted for spatial autocorrelation explicitly and reduced un-modeled spatial dependence. The neighbors for each municipality were obtained from a shapefile for Brazil.

We also used a nonparametric random walk of order two temporal model with no space-time interaction. Lastly, we included type I space-time interaction, which assumed interaction between the unstructured effects (Blangiardo and Cameletti 2015). The reported results were for the model with the smallest estimation error as measured by the Deviance Information Criterion (DIC). We reported 95% credible intervals which represent the 2.5th to 97.5th percentiles of the posterior distribution of estimated homicides rates.

We fitted all models using an integrated nested Laplace approximation (INLA) approach available through the R-INLA package (Blangiardo and Cameletti 2015; Lindgren and Rue 2015; R Core Team 2013). INLA is a computationally efficient technique that has been shown to be extremely accurate and more efficient compared to Markov Chain Monte Carlo Methods (MCMC) (Blangiardo and Cameletti 2015). It is designed for latent Gaussian models, which include a wide class of spatial and spatiotemporal models. Default, non-informative priors were used.

## Results

**Fig. 1:**
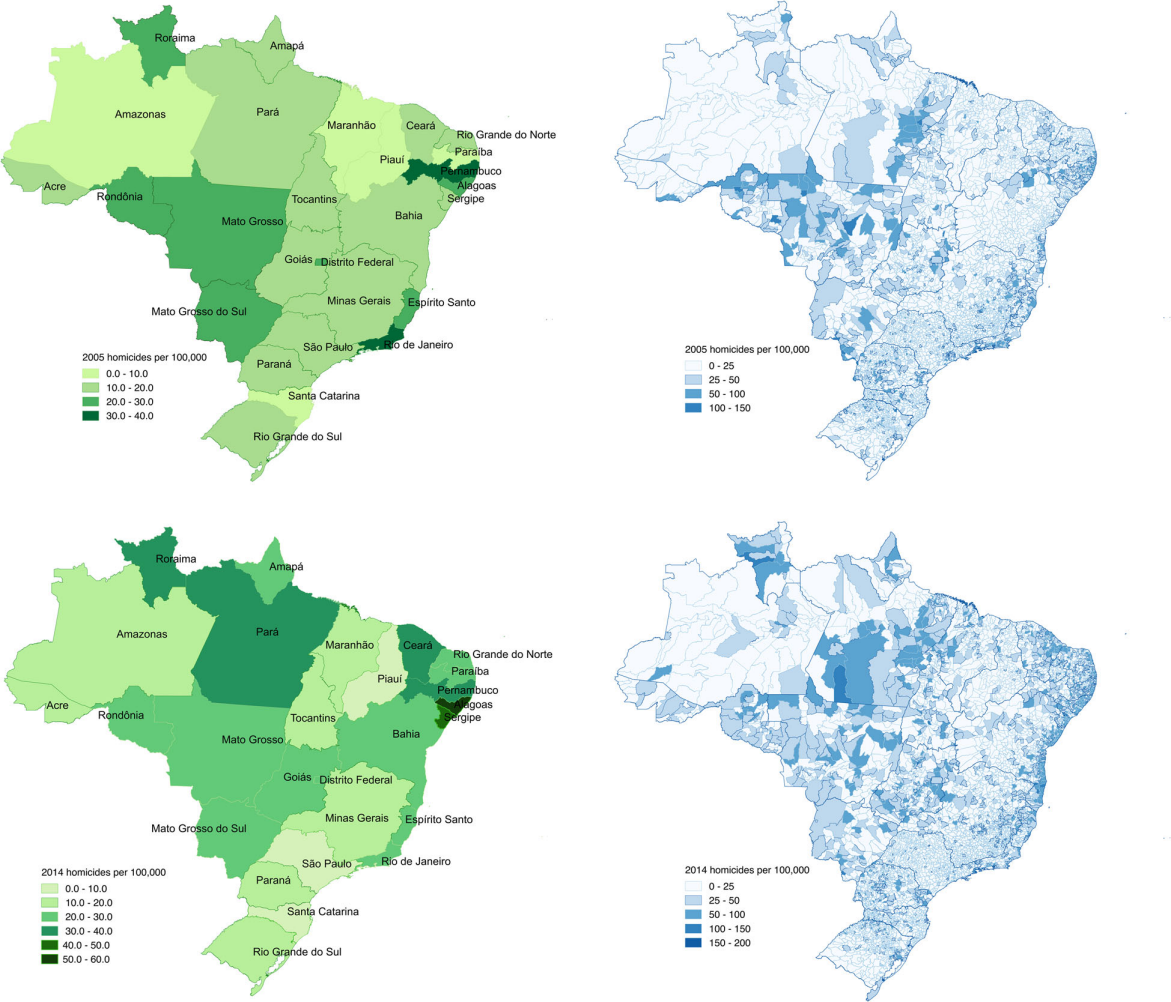
Mean yearly homicide rate per 100,000 at the state level (**a**) and (**b**), and the municipality level (**c**) and (**d**) for 2005 and 2014, respectively

Average homicide rates varied across municipalities and states within our study period (Fig. 1a and b). For example, the neighboring northeastern states of Pernambuco and Alagoas had the highest average homicides; 33 and 34 homicides per 100,000 persons, respectively. These values doubled in 2014 (51 per 100,000 persons) compared to 2005 (25 per 100,000 persons) for Alagoas. In contrast, the Santa Catarina and Piaui states had the lowest reported average homicide rates during the study period; 8 and 6 homicides per 100,000 persons, respectively. However, not all spatial trends were replicated at the municipality level. Most municipalities with an increase in homicide rates were clustered in the central, northeastern and eastern municipalities along the coast (Fig. 1c and d).

**Fig. 2:**
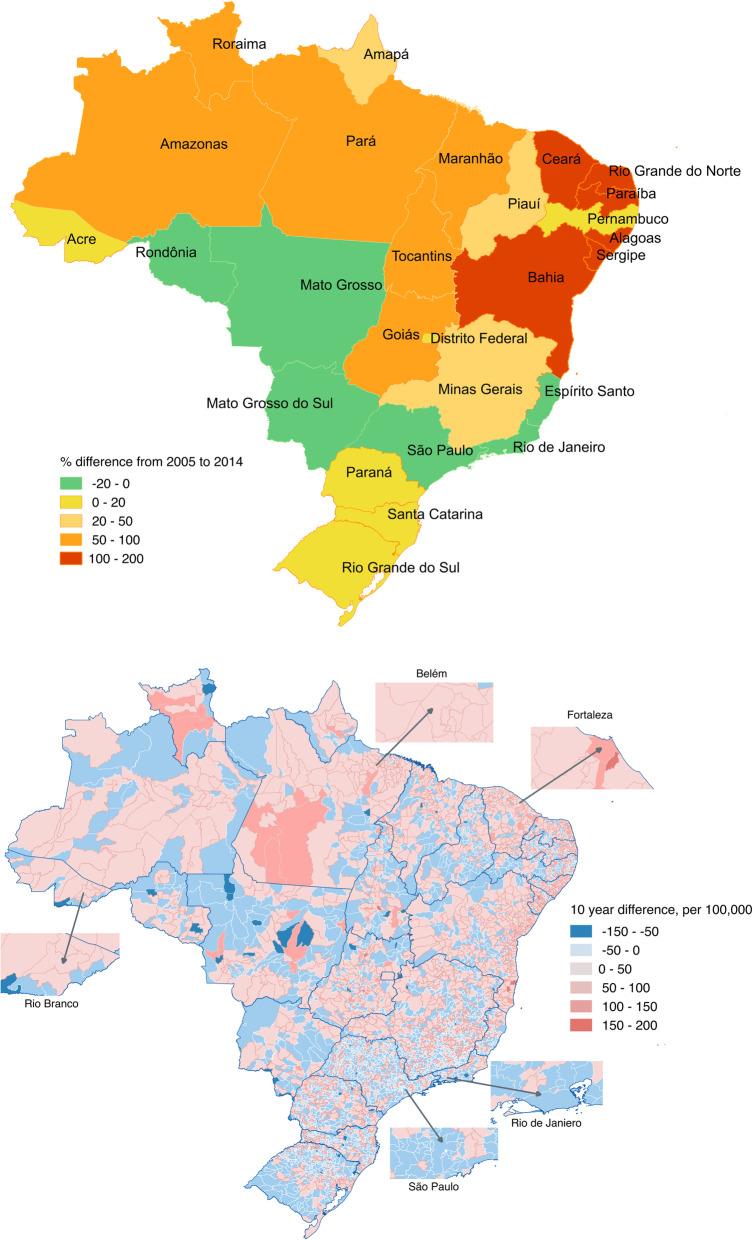
The change in homicide rates over time for states (top) and municipalities (bottom) in Brazil

We noted the largest decrease in homicide rates in the western and southeastern states of Sao Paulo, Rio de Janeiro and Espirito Santo (Fig. 2). This decline coincided with an increase in homicide rates in the northeastern states of Ceará, Alagoas, Paraiba, Rio Grande Norte, Sergipe and Bahia. However, not all municipalities within states with an overall decrease also noted a decrease in homicide rates. For example, while homicide rates declined in municipalities such as Sao Paulo and Rio de Janeiro, some neighboring municipalities saw an increase.

**Fig. 3:**
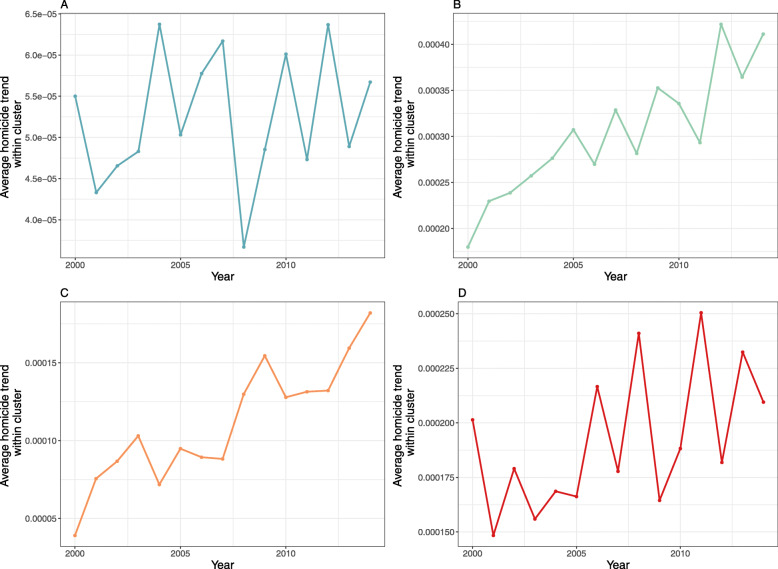
Homicide trends for municipalities with population less than 25 thousand clustered into four groups using principal component cluster analysis. Each curve represents the average trend within each cluster. Each cluster contains the following number of municipalities: A. 1765 (42.1%), B. 554 (13.2%), C. 1177 (28.1%) and D. 695 (16.6%). The overall trend suggests an increase in homicide rates, which supports our study hypothesis that homicide rates have increased in municipalities with smaller populations

**Fig. 4:**
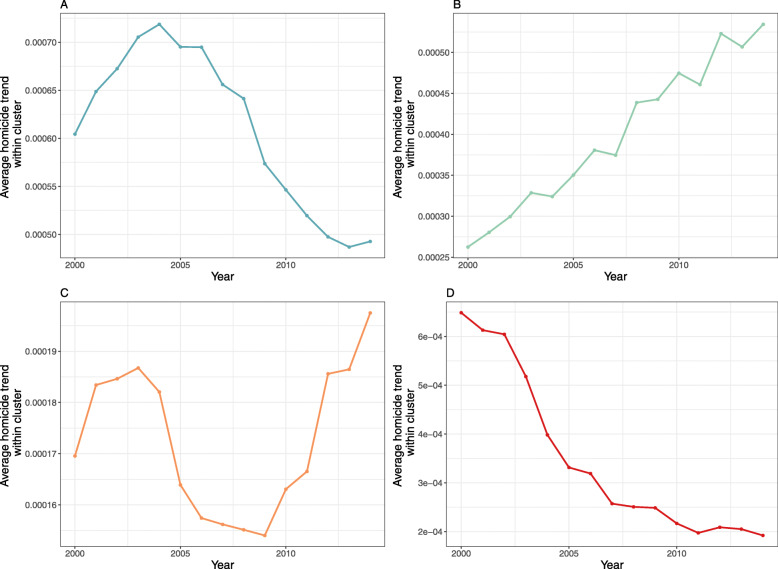
Homicide trends for municipalities with populations of at least 250 thousand clustered into four groups using principal component cluster analysis. Each curve represents the average trend within each cluster. The number of municipalities in each cluster are: **a**. 21 (19.1%), **b**. 32 (29.1%), **c**. (30.0%) and **d**. 24 (21.8%). The homicide rates show an overall decreasing trend for some municipalities, and an increasing trend in others

The PCA clustering to identify municipalities with similar temporal trajectories in homicide rates showed an overall increasing trend in homicide rates across municipalities with populations of 25,000 or less, from 2000 to 2014. The trend for each of the clusters was upwards over the study period (Fig. 3). Municipalities with population sizes between 25,000 and 100,000 also displayed an increasing trend in homicide rates for most municipalities (SI Fig. 1). In contrast, homicide rates on average decreased in municipalities with a population size of 250,000 people or more, and two of the four trajectories trend steeply downwards (Fig. 4).

The change in homicides between municipalities was similar to the change between states across the study period (Table 2). The risk of homicides was higher if the municipality was a metro region (Relative Risk (RR), 1.44, 95% CI, 1.38–1.52), bordered a country strip (1.151, 95% CI, 1.08–1.23), had a higher crude domestic product (1.18, 95% CI, 1.15–1.21) and a high proportion of the population aged 15–29 (1.07, 95% CI, 1.06–1.08). These findings were robust to another specification of the country border variable.

**Table 2:** Posterior mean and 95% credible intervals

	Space-time model
Intercept	−12.337 (−12.650, −12.023)
Log crude domestic product	0.168 (0.143, 0.192)
Aged (15–29) proportion	0.069 (0.062, 0.075)
Border countries strip	0.141 (0.076, 0.205)
Metro region	0.370 (0.324, 0.417)
Municipality variance	0.269 (0.267, 0.276)
State variance	0.201 (0.197, 0.206)

## Discussion

Our findings support both the dissemination hypothesis and the interiorization hypothesis. Consistent with dissemination, we observed stark increases in homicide rates in Northeastern and Midwest states at the same time as decreases in Southeastern states. Consistent with interiorization, we saw large heterogeneity in the temporal trends of large municipalities but increases across all trajectory clusters for small municipalities. Homicide rates in most state capitals (especially the largest ones - Rio de Janeiro and São Paulo) have stabilized while increasing in the smaller municipalities. Our spatiotemporal model also indicated that these effects may be explained by structural factors including crude domestic product, metro regions, municipalities bordering neighboring countries and proportion of population aged fifteen to twenty-nine.

While these findings are consistent with prior work (Soares Filho et al. 2020; Scorzafave et al. 2015), ours is the first to perform a spatial and temporal assessment over this fifteen-year period to show shifts in homicide rates across Brazil and investigate the two important hypotheses; dissemination and interiorization. We found that proximity to a national border was a significant predictor of changes in the homicide rates. This finding is suggestive of the role played by drug and weapons trafficking in local homicide rates. Previous research has found similar effects in other South American countries. For instance, a study on homicide and youth in the state of Santa Catarina and Paraná in Paraguay showed an increase in homicide rate in the cities located on country borders and those close to highways that connect major cities in South and South East regions of Brazil and Paraguay (Wanzinack et al. 2018). Furthermore, the North, Northeast, and Central regions of Brazil which are agricultural frontiers with land tenure-related conflicts have the highest homicide rates. These regions have had continuous conflicts over land (Reichenheim et al. 2011). In contrast, the South and South East regions, which have higher population density and are more developed economically, have the lowest homicide rates.

These findings highlight how major changes in economic development and land use, as have occurred in Brazil since 1990, can contribute to changes in fatal violence. National violence prevention strategies must account for these trends in order to allocate resources appropriately. Moreover, the presence of spatial diffusion, i.e. dissemination, indicates that focused prevention efforts in one location may produce indirect benefits for nearby locations by preventing or reversing the transmission of violence through space. As Brazil implements new national strategies for preventing violence, evaluations should consider the possible spatial spillover effects of interventions focused in particular areas (Brännström et al. 2016). Our findings also have implications for the content of those programs. We found that conditional on gross domestic product, poverty was positively associated with firearm homicides at the municipality level. In other words, violence increased as economies grew and community members were left behind.

Our findings suggest policies should, (a) address the growth of poverty that occurs alongside economic development and (b) consider possible spatial spillover benefits from focusing interventions at a time/place where violence is increasing. Urban violence is known to be multi-causal. It is partly based on the scarcity or inefficiency of the State’s presence; precarious social and public security policies and scarce access to justice. It is enhanced by social inequalities reflected in the high rates of school dropout, illiteracy and unemployment, access to and consumption of alcohol and drugs, availability of weapons, group conflicts, domestic violence and family breakdown (Caicedo et al. 2010; Gorman et al. 2018; Drumond at al, 2015; Murray et al. 2013). The confrontation of urban violence requires intrasectoral, coordinated and continuous actions, directed to its most diverse etiologies and not only to its more superficial consequences. Further studies to elucidate the reasons for the trends observed in this study can greatly assist the effectiveness of actions taken to address the growth of violence in small and medium sized Brazilian municipalities. Homicide is a serious social problem with intense repercussions for the health sector, and should be a recurrent object of analysis and public policies.

Additionally, the recent economic crisis and loss of financial capacity of the Brazilian State, make social and security policies increasingly fragile, and may have an important impact on the trend and intensity of the risk of homicide in different spaces. Furthermore, the credibility crisis arising from corruption and the precarious governance affect the sustainability of public policies (Barbosa Filho 2017). The understanding of violence in this new context, its mobility and associated factors demands investigations that can point out conglomerates and priority events in specific scenarios to enable the implementation of fundamental policies, which can be reproducible and sustainable in various locations.

There are limitations in our analysis. The covariates used in our analysis were not all available at the municipality level, this implies that spatial differences across municipalities were lost when aggregated at the state level, which could impact the model outcomes. Our analysis also assumes the data is accurate. However, differences in state surveillance systems can lead to variabilities in reporting. Additionally, different ethnicities are impacted differently by homicides (Tavares et al. 2016), however, we could not incorporate ethnicity in our analysis because definitions are not consistent and the variable race in our data was largely ignored or left blank. Lastly, there are other covariates (e.g., alcohol abuse, gun ownership) that were not available for all the years and were therefore not included in the analysis.

## Conclusions

Our study provides important findings that describe longitudinal spatial and temporal changes in homicide rates across municipalities and states. Our findings support both a displacement hypothesis and an interiorization hypothesis. An understanding of how different factors are contributing to these changes can aid in the development of policies aimed at reducing homicides in Brazil.

## Supplementary information

**Additional file 1: SI Figure 1.** Homicide trends for municipalities with population between 25 and 100 thousand clustered into four groups using principal component cluster analysis. Each curve represents the average trend within each cluster. Each cluster contains A. 477 (44.4%), B. 212 (19.7%), C. 106 (9.9%) and D. 279 (26.0%) municipalities. The homicide rates show an overall increasing trend, which supports our hypothesis of increase in homicide rates for municipalities with smaller populations. **SI Figure 2:** Homicide trends for municipalities with population between 100,000 and 250,000 thousand clustered into four groups using principal component cluster analysis. Each curve represents the average trend within each cluster. Each cluster contains A. 34 (17.9%), B. 53 (27.9%), C. 73 (38.4%) and D. 30 (15.8%) municipalities. The homicide rates are increasing for some municipalities and decreasing in others.

## Data Availability

Data is available from the Brazil Ministry of Health and can be provided to researchers upon request.
