# Supplementary material for: Mapping disparities in homicide trends across Brazil: 2000–2014
Source: Inj Epidemiol. 2020 Sep 7;7:47. doi: 10.1186/s40621-020-00273-y (PMC7487619; doi:10.1186/s40621-020-00273-y)
Supplement: Supplementary file 1 — Additional file 1: SI Figure 1. Homicide trends for municipalities with population between 25 and 100 thousand clustered into four groups using principal component cluster analysis. Each curve represents the average trend within each cluster. Each cluster contains A. 477 (44.4%), B. 212 (19.7%), C. 106 (9.9%) and D. 279 (26.0%) municipalities. The homicide rates show an overall increasing trend, which supports our hypothesis of increase in homicide rates for municipalities with smaller populations. SI Figure 2: Homicide trends for municipalities with population between 100,000 and 250,000 thousand clustered into four groups using principal component cluster analysis. Each curve represents the average trend within each cluster. Each cluster contains A. 34 (17.9%), B. 53 (27.9%), C. 73 (38.4%) and D. 30 (15.8%) municipalities. The homicide rates are increasing for some municipalities and decreasing in others. [file 40621_2020_273_MOESM1_ESM.docx]

**Supplementary Information**

**Mapping Disparities in Homicide Trends across Brazil: 2000-2014**

Elaine O. Nsoesie^1^, Antonio S. Lima Neto*^2,3,4^, Jonathan Jay*^5,6^, Hailun Wang^1^, Kate Zinszer^7^, Sudipta Saha^8^, Adyasha Maharana^9^, Fatima Marinho^10^, Adauto Martins Soares Filho ^10^

1. Department of Global Health, School of Public Health, Boston University, Boston, Massachusetts, United States
2. Fortaleza Municipal Health Secretariat (SMS-Fortaleza), Fortaleza, Ceará, Brazil.
3. University of Fortaleza (UNIFOR), Fortaleza, Ceará, Brazil
4. Takemi Program, Harvard T.H. Chan School of Public Health, Boston, Massachusetts, United States
5. Firearm-safety Among Children and Teens Consortium, University of Michigan School of Medicine, Ann Arbor, Michigan, United States
6. Department of Health Policy & Management, Harvard T. H. Chan School of Public Health, Boston, Massachusetts, United States
7. Department of Social and Preventive Medicine, University of Montreal, Montreal, Quebec, Canada
8. Department of Global Health and Population, Harvard T. H. Chan School of Public Health, Boston, Massachusetts, United States
9. Department of Computer Science, University of North Carolina, Chapel Hill, NC, USA
10. Department of Health Surveillance, Ministry of Health, Brasilia, Brazil

**SI Figure 1:** Homicide trends for municipalities with population between 25 and 100 thousand clustered into four groups using principal component cluster analysis. Each curve represents the average trend within each cluster.  Each cluster contains A. 477 (44.4%), B. 212 (19.7%), C. 106 (9.9%) and D. 279 (26.0%) municipalities. The homicide rates show an overall increasing trend, which supports our hypothesis of increase in homicide rates for municipalities with smaller populations.

**SI Figure 2:** Homicide trends for municipalities with population between 100,000 and 250,000 thousand clustered into four groups using principal component cluster analysis. Each curve represents the average trend within each cluster.  Each cluster contains A. 34 (17.9%), B. 53 (27.9%), C. 73 (38.4%) and D. 30 (15.8%) municipalities. The homicide rates are increasing for some municipalities and decreasing in others.
